# Polymer Waveguide Sensor Based on Evanescent Bragg Grating for Lab-on-a-Chip Applications

**DOI:** 10.3390/s24041234

**Published:** 2024-02-15

**Authors:** Zhenyu Zhang, Ahmad Abdalwareth, Günter Flachenecker, Martin Angelmahr, Wolfgang Schade

**Affiliations:** 1 Department for Fiber Optical Sensor Systems, Fraunhofer Heinrich Hertz Institute, 38640 Goslar, Germany; zhenyu.zhang@hhi.fraunhofer.de (Z.Z.); ahmad.abdalwareth@hhi.fraunhofer.de (A.A.); martin.angelmahr@hhi.fraunhofer.de (M.A.); wolfgang.schade@hhi.fraunhofer.de (W.S.); 2 Institute of Energy Research and Physical Technologies, Clausthal University of Technology, 38640 Goslar, Germany

**Keywords:** optical sensor, Bragg grating, polymer waveguide, lab-on-a-chip, on-chip optical sensor, evanescent field sensor, nanoparticles, hydrogen sensor

## Abstract

In this work, an evanescent Bragg grating sensor inscribed in a few-mode planar polymer waveguide was integrated into microchannel structures and characterized by various chemical applications. The planar waveguide and the microchannels consisted of epoxide-based polymers. The Bragg grating structure was postprocessed by using point-by-point direct inscription technology. By monitoring the central wavelength shift of the reflected Bragg signal, the sensor showed a temperature sensitivity of −47.75 pm/K. Moreover, the functionality of the evanescent field-based measurements is demonstrated with two application examples: the refractive index sensing of different aqueous solutions and gas-phase hydrogen concentration detection. For the latter application, the sensor was additionally coated with a functional layer based on palladium nanoparticles. During the refractive index sensing measurement, the sensor achieved a sensitivity of 6.5 nm/RIU from air to 99.9% pure isopropyl alcohol. For the gas-phase hydrogen detection, the coated sensor achieved a reproducible concentration detection up to 4 vol% hydrogen. According to the reported experimental results, the integrated Bragg-grating-based waveguide sensor demonstrates high potential for applications based on the lab-on-a-chip concept.

## 1. Introduction

Devices based on lab-on-a-chip (LOC) technology offer a variety of miniaturized fluidic and nonfluidic systems for chemical and biological applications [1]. LOC devices possess the advantages of a miniaturized size, fast response with small volumes of samples and low-cost analysis [2]. During the past few decades, the potential of LOC devices has been widely explored with various microengineered sensor platforms embedded in a device that allows various chemical or biological measurements [3]. In this case, optical sensors, especially Bragg-grating-based sensors, due to their small size and low power requirements for operation, have driven increasing attention as they can be integrated as a sensor element in LOC devices [4,5].

Bragg gratings are periodic spatial modulations of the refractive index along the optical waveguide. One of the most common Bragg grating sensors are fiber Bragg gratings (FBGs) inscribed with a laser inside a silica-based fiber core [6]. Since first introduced by Kawasaki et al. in 1978 [7], FBGs have been widely investigated for indirect measurements in physical and chemical areas [8]. However, the protective sheath of the conventional fibers limits the direct interaction of the guided optical mode with the surrounding medium. To enable an evanescent interaction with the FBG sensors, a chemical etching process is often required to remove the cladding of the silica fiber. The resulting etched fiber Bragg grating (eFBG) sensor can be directly applied for measuring various refractive indices from different liquid solutions [9]. Moreover, a LOC device integrated with an eFBG sensor was presented by Lee et al. for measuring the microstrain, temperature and refractive indices [10].

Nevertheless, optimizations can still be made to further increase the stability and sensitivity of the integrated Bragg grating sensor in both fabrication and measurement processes. For instance, through the etching process, the eFBG signal may shift from the original addressed Bragg wavelength and intensity and, in extreme cases, alter the original signal completely [11]. These possible variations during the etching process typically result in a lowered reproducibility of the eFBG sensors. Furthermore, since polymers are one of the commonly used materials for LOC devices, eFBGs cannot be embedded in LOC devices consisting of polymer materials that possess a greater refractive index than the fiber core; otherwise, the light transmitted through the fiber core will be completely coupled into the surrounding polymers, resulting in the loss of the signal. To overcome these disadvantages, polymer planar optical waveguide sensors could be a better alternative replacing etched silica-based fiber for LOC device integration. Polymer waveguides with a Bragg grating structure were directly induced with lasers on the surface of a polymeric substrate by Wochnowski et al. [12] and Kefer et al. [13]. The former work reported that their laser induction process led to a rougher surface where the waveguide was located. However, this could limit the positioning of further LOC structures, which subsequently are applied to the substrate’s surface. Instead, photolithography technologies offer a more flexible fabrication of polymer waveguides and the LOC structure within the same production process. For example, Meyer et al. presented a polymer waveguide patterned by a lithography machine and inscribed with Bragg grating structures, and the sensor showed an increased sensitivity compared to FBGs in temperature and strain [14]. However, their presented polymer waveguide Bragg grating sensor was not directly applicable for evanescent field detection as they used a polymer cladding, shielding the waveguide from the surrounding media. A polymer waveguide without cladding would enable the evanescent wave interaction and opens more applications like refractive index sensing and more precise chemical detection capabilities coated with functional layers.

Functional layers are commonly used for optical sensors to achieve a selective detection of specific gases [15,16]. This possibility makes optical sensors attractive as general sensor platforms, which can be functionalized individually depending on the target molecules to be detected. For example, Kefer et al. presented a laser-infused evanescent polymer waveguide Bragg grating sensor coated with Pt-loaded WO_3_-SiO_2_, which achieved a detection limit of 5 ppm hydrogen in air [13]. This methodology provides a possibility for optical hydrogen sensors implementable in LOC devices and provides great potential for researchers investigating the underground biomethanation reactor concept, in which hydrogen and carbon dioxide are converted into methane inside underground porous formations. This concept has been attracting increasing attention in the high-demanding hydrogen energy research field in recent years [17]. However, a wider sensor dynamic range than a ppm area for hydrogen detection is desirable for specific applications like analyzing the efficiency of the methanation reaction [18]. In this case, most recently, Abdalwareth et al. presented an eFBG sensor coated with palladium nanoparticles and achieved a hydrogen detection up to 5 vol% [19]. It is most interesting to investigate if the same coating can be applied on a planar polymer waveguide sensor based on Bragg grating structures.

In this work, we present an evanescent Bragg grating sensor based on the polymer waveguide, which was manufactured simultaneously alongside the LOC structure by using the same processing steps. The air-cladded few-mode ridge waveguide consisted of epoxide-based polymers. The waveguide was patterned with epoxy photoresist applied on a silica glass substrate by using photolithography technology. The Bragg grating structure was inscribed subsequently into the waveguide by a femtosecond laser as part of a separate processing step. The temperature dependence of the ridge waveguide Bragg grating (RWBG) sensor was characterized for different ambient temperatures from 20 °C to 60 °C. The influence of the environmental humidity on the polymeric waveguide-based RWBG was also characterized and discussed. Further, the performance of the sensor for the detection of chemicals was validated and demonstrated by both the liquid-phase refractive index measurement with different concentrated isopropyl alcohol (IPA) solutions as well as gas-phase hydrogen detection. For hydrogen detection, the sensor was additionally coated with palladium nanoparticles.

## 2. Materials and Methods

### 2.1. Waveguide Fabrication

**Figure 1:**
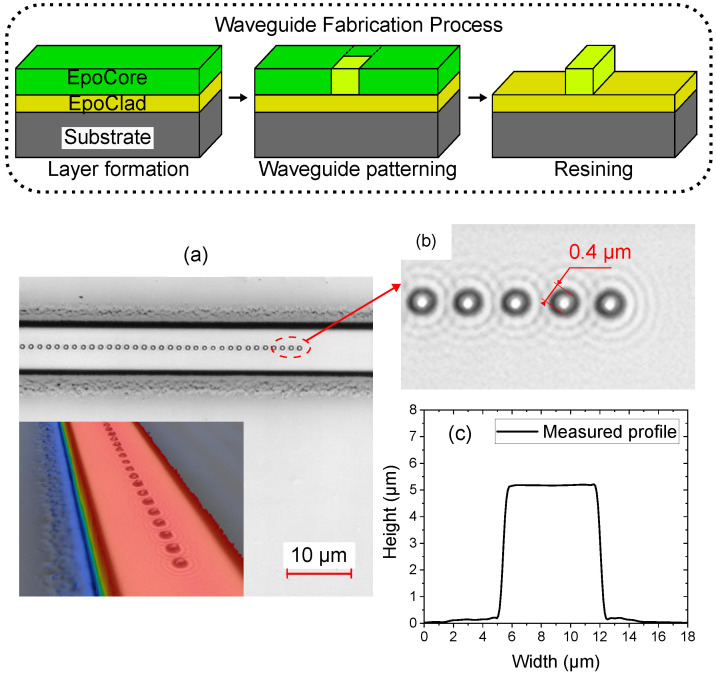
Scheme (inside dashed line area) displaying the fabrication process of the waveguide and the resulting waveguide measured with LSM. (**a**) Microscopic photo of the RWBG (inset: 3D height profile). (**b**) An enlarged area demonstrating the laser-inscribed Bragg grating structure. (**c**) Cross-section profile of the polymer ridge waveguide.

The fabrication process begins with an oxygen plasma treatment for the glass substrate to create a hydrophilic surface for optimal adhesion. After the oxygen treatment, a substrate layer for the waveguide, consisting of EpoClad2 from microresist technology GmbH, is deposited on the oxygen-activated surface of the substrate with a spin-coating process. The substrate polymer has a refractive index of 1.57 at 830 nm, which is lower than the polymer EpoCore5 (a refractive index of 1.58 at 830 nm) used for patterning the waveguide [20]. The substrate layer aims to ensure the optical confinement of the guided light in the waveguide that is applied on top of this substrate layer. The layer thickness of the polymers depends on the spin-coater speed, and here the substrate polymer is adjusted to 2 µm. After the spin-coating process, the glass substrate with the EpoClad2 layer was prebaked for 5 min at 120 °C. Subsequently, after applying flood exposure with UV light, the polymer substrate was hard-baked on a heat plate at 120 °C for 30 min. After the polymer substrate was prepared, its surface was again activated with oxygen plasma. Immediately after this, we added a 5 µm layer of EpoCore5 on top of it via spin coating. The substrate with both layers was then prebaked on a heat plate subsequently at 50 °C for 2 min and 90 °C for 4 min. Afterward, we applied a waveguide pattern by using a UV-photolithography machine (µPG 101, Heidelberg instruments, Heidelberg, Germany). Consequently, the substrate with patterned polymers was postbaked on the heat plate with the same parameters from prebake processes. Finally, the substrate with polymer layers was immersed in the developer (mrDev600, micro resist technology GmbH, Berlin, Germany) for 3 min to resin the remaining unexposed polymers. A final hard-bake process on the heat plate at 120 °C for 30 min concluded the fabrication process for the ridge waveguide, and thus the waveguide was then ready for Bragg grating inscription. Inside the dashed line area of Figure 1, a scheme of the fabrication process is displayed. The waveguide enlarged with a 100× objective and its cross-section profile measured with a laser scanning microscope (LSM) (VK-X250, Keyence Deutschland GmbH, Neu-Isenburg, Germany) is displayed in Figure 1c; the cross-section profile of the waveguide shows a dimension of 6.5 µm width and 5 µm height.

The cross-section profile size of our polymer waveguides was limited by the accuracy of the photolithography machine. Therefore, the presented parameters aim to ensure an optimal fabrication reproducibility of the resulting waveguide. The cross-section waveguide profile slightly exceeds the overall size of a single-mode polymer waveguide by using the same EpoCore material presented by Elmogi et al. with 5 µm in width and height [21]. Therefore, a few-mode light propagation inside our presented waveguide is possible. However, the waveguide is coupled to a single-mode fiber for a signal read-out, which means only the fundamental mode is excited. We assume that, within the short length of the straight waveguides, the fundamental mode is preserved.

### 2.2. Bragg Grating Inscription

We applied femtosecond laser pulses to achieve uniform periodic refractive index modulations along the polymer waveguide. Such Bragg grating structures are spectral small band mirrors with a central reflection wavelength $\lambda_{B}$ [6]:

(1) $$\lambda_{B}=\frac{2n_{eff}\Lambda }{m},$$

where $n_{eff}$ is the effective refractive index, Λ represents the grating period and *m* is the grating order determined as a natural number (m= 1, 2, 3, …). In this work, the grating order *m* is determined as 5 to avoid the overlapping of grating points during the inscription process while ensuring the optimal reproducibility of the measured Bragg signal. For a central wavelength $\lambda_{B}=$ 850 nm, the period of the grating points Λ is calculated to be 1.43 µm for the 5th grating order.

The setup for Bragg grating inscription consists of a commercially available femtosecond laser (Ti/sapphire Tsunami/Spitfire pro, Newport Spectra-Physics GmbH, Darmstadt, Germany). The laser pulses had a central wavelength of 800 nm and the pulse duration was 85 fs [22]. The substrate with the ridge waveguide was fixed on three translation stages (N-565.260, N-565.360 and E-709, Physik Instrumente, Karlsruhe, Germany) to enable high-precision three-dimensional movements during the Bragg grating inscription process. During this process, the laser pulses were focused by a 20× objective lens (LD Plan-Neoflur 20×, Carl Zeiss AG, Oberkochen, Germany), and the energy of the laser was set at 29 nJ at a repetition of 10 Hz measured under an objective lens. A detailed picture of the Bragg grating taken by LSM is displayed in Figure 1a. The inset picture in Figure 1a shows a three-dimensional profile of the waveguide including the laser-inscribed Bragg grating structure, measured by LSM. Figure 1b shows a top view of the refractive index modulation points caused by single laser pulses, and the average diameter of the grating points was 0.4 µm as a result of applying the 20× focusing objective. The total length of the Bragg grating structure was 3 mm.

### 2.3. Sensor Integration in LOC Concept

**Figure 2:**
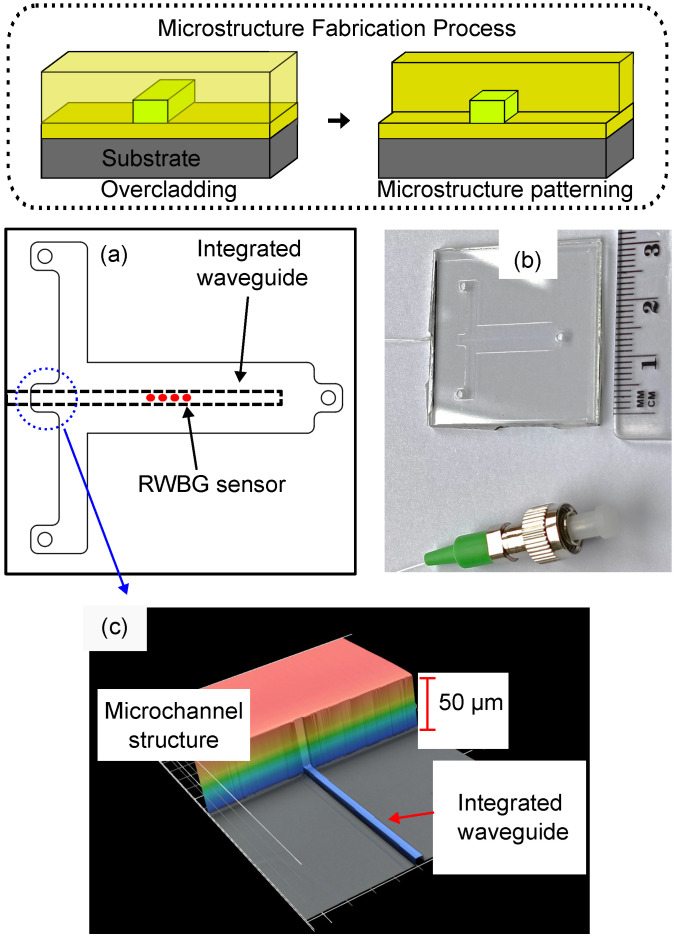
Scheme (inside dashed line area) displaying the fabrication process of the microstructure and the T-junction structure for LOC sensor integration. (**a**) Sketch of the T-junction design and positioning of the waveguide and RWBG. (**b**) Photo of the manufactured RWBG sensor chip. (**c**) 3D height profile of the T-junction: The 5 µm high waveguide channels through a 50 µm high microstructure.

In this work, we designed a T-junction microchannel structure to guide test fluids onto the sensor position; the overall size of the microchannel structure is 30 mm in square, and its sketch is displayed in Figure 2a. The designed structure consists of one broader microchannel that holds the waveguide and two narrower microchannels enabling the external injection of liquids into the main channel. On the tips of all three microchannels are inlet positions that allow for the connection of the external fluid injection or can be used as a waste outlet. In this concept, the RWBG will serve as an optical sensor element, enabling the in situ measurement of refractive indices from liquid solutions.

The fabrication of the T-junction structure took place directly after the Bragg grating inscription. It began with the application of an additional polymer resist layer of EpoClad50 (with a 50 µm layer thickness) onto the RWBG sensor surface. After the polymer deposition was finished using the spin-coater, the T-junction structure was patterned by the same photolithography machine (see Section 2.1). In Figure 2c, a 3D laser scanning height profile of the place is shown, where the ridge waveguide tunnels through the higher microchannel boundary.

Lastly, the sealing of the T-junction structure took place by adhering another polymer-applied glass substrate as the top seal of the microchannels, forming a compact LOC device. The sealing glass substrate includes boreholes on positions where the corresponding inlet channels from the T-junction structure are located. Another EpoClad50 layer with a layer thickness of 50 µm was applied on the surface of the sealing glass substrate to serve as a bonding polymer to adhere to the microchannel layer. Afterward, the two substrates were pressed together, followed by a hard-bake process, completing the fabrication for a RWBG integrated compact LOC device. A scheme of the T-junction structure fabrication process and the fabricated LOC device is displayed in Figure 2b. The optical read-out of the RWBG was realized by butt coupling a single-mode glass fiber to one open end of the waveguide at the edge of the LOC sensor chip by using a 3-axis positioning stage (MAX313D/M, Thorlabs, Inc., Bergkirchen, Germany) and fixed onto the end of the waveguide with UV adhesive (PO-67-LS, Dymax Europe GmbH, Wiesbaden, Germany).

### 2.4. Functional Coating for Hydrogen Detection

**Figure 3:**
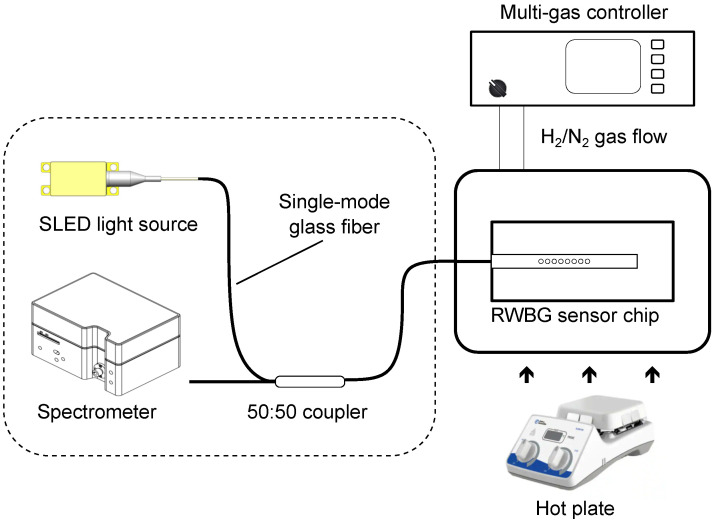
Optical measurement setup and read-out unit.

Aside from being used as an evanescent sensor in liquid-phase refractive index measurements, the RWBG sensor is also capable of the selective sensing of gases by applying a functional coating. Here, we demonstrate this functionality concept by using palladium nanoparticles as the functional coating for selective hydrogen gas detection. For the preparation of the coating process, we exposed the surface of the RWBG sensor to oxygen plasma to ensure a hydrophilic surface. Then, we wetted the sensor with drops of a dissolved palladium nanoparticles solution until the whole grating area was fully covered. The palladium nanoparticles (HiQ-Nano s.r.l., Arnesano, Italy) used here possess an average size of 8 nm and a size distribution between 7 nm and 9 nm, and the nanoparticles were dissolved in distilled water with a concentration of 1 mg/mL. This coating process was monitored in real time with the optical measurement setup presented in Figure 3. Finally, the intensity of the RWBG reflection signal decreased up to half of the original position. In the meantime, the palladium nanoparticle solution on the sensor surface was visually vaporized.

### 2.5. Optical Measurement Setup

To evaluate the read-out signal of the RWBG, a broadband SLED with a center wavelength of 840 nm (EXS210006-01, EXALOS AG, Schlieren, Switzerland) was used as the light source, the emitted light beam was coupled through a 50:50 fiber coupler and transmitted into the RWBG, and finally, the back-reflected Bragg signal was coupled via the fiber coupler into a spectrometer (Flame-S-VIS-NIR, Ocean Insight). The optical measurement setup is illustrated in Figure 3 inside the dashed line area. As the RWBG sensor chip was connected only via a single glass fiber to the interrogator, the flexibility of handling the chip in different experimental setups was high. To test the sensor at different temperature conditions, the RWBG sensor chip was placed on a tunable hot plate (Fisherbrand Isotemp, Thermo Fisher Scientific GmbH, Dreieich, Germany). For the gas-phase hydrogen measurement, the sensor chip was enclosed in an experimental gas chamber with a connection to the gas flow controller system (647C, MKS Instruments GmbH, Munich, Germany) to adjust different concentrations of the gas mixtures.

### 2.6. Sensing Principles

The principle of temperature sensing with the presented RWBG sensor is based on the Bragg condition expressed in Equation (1). The effective refractive index $n_{eff}$ and the grating period Λ are defined during the Bragg grating inscription process, resulting in a narrow reflection spectrum that can be measured with the presented measurement setup. Changes from the environmental temperature ΔT affect a shift in the Bragg wavelength according to

(2) $$\Delta \lambda_{B}=\lambda_{B}\left(\alpha_{\Lambda }+\alpha_{n}\right)\Delta T,$$

here, $\alpha_{\Lambda }$ is the coefficient of thermal expansion of the polymer material and $\alpha_{n}$ is the thermo-optic coefficient. The temperature dependence of the Bragg sensor can be evaluated by monitoring the central wavelength shift of the reflected Bragg signal caused by temperature change.

Additionally, as the presented waveguide was not covered with an additional polymer cladding, a part of the propagating light mode can directly interact with the ambient medium through its evanescent field. Therefore, if the refractive index of the ambient medium changes, the effective refractive index $n_{eff}$ is also changing; hence, a shift in the reflected central Bragg wavelength $\Delta \lambda_{B}$ is induced. This functionality can be mathematically expressed by [23,24]:

(3) $$\Delta \lambda_{B}=\frac{2\Delta n_{eff}\Lambda }{m},$$

In this case, the shift in the reflected central Bragg wavelength $\Delta \lambda_{B}$ is the measuring quantity to investigate environmental refractive index changes.

Finally, the functionality of the evanescent wave hydrogen sensor based on RWBG is realized by applying an additional functional coating of palladium nanoparticles introduced in Section 2.4. Structural changes in the coating, induced by hydrogen, can be detected via the evanescent interaction of the guided light wave with the coating. Since palladium exhibits a high optical absorption coefficient, it will initially lead to a high optical loss during the coating process. When exposed to hydrogen, palladium rapidly converts into reversible palladium hydride, resulting in changes in the evanescent field absorption from the functional coating as well as the transmitted optical signal intensity [25]. When the palladium functional coating is applied to the presented RWBG sensor, variations in the hydrogen content are monitored by the change in the Bragg signal intensity.

## 3. Results

### 3.1. RWBG Reflection Spectrum and Temperature Dependence

**Figure 4:**
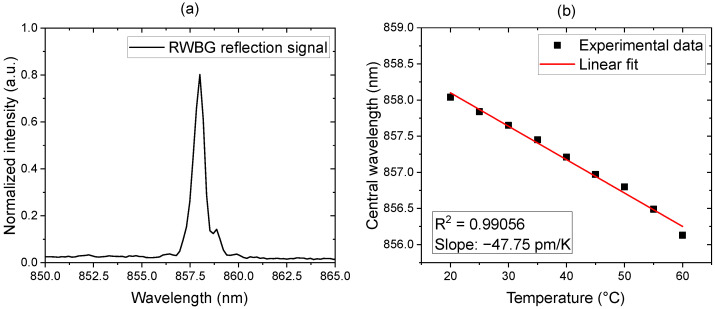
(**a**) Reflection spectrum of the RWBG. (**b**) Temperature-dependence-measurement result.

The reflection spectrum of the RWBG, achieved with the fiber-coupled measurement setup shown in Figure 3, is displayed in Figure 4a. According to the manufacturer, the optical loss of the EpoCore polymer is 0.2 dB/cm [20]. The butt-coupling point where the polymer waveguide was connected to a single-mode glass fiber via UV adhesive can typically result in about a 0.5 dB connection loss. The total optical loss of the LOC device is assumed to be 2 dB.

To evaluate the temperature sensitivity of RWBG, the sensor chip was placed on a hot plate. We started the measurement at room temperature (20 °C with a 40% humidity level). Subsequently, the temperature of the hot plate was increased stepwise by 5 °C until a temperature of 60 °C was reached. During the experiment, the air humidity level was kept constant at 40%, and the central wavelength of the reflected Bragg signal was monitored continuously at each temperature level. The measurement results are shown in Figure 4b. As can be seen from these measurement data, the wavelength of the Bragg reflection is decreasing linearly with a slope of −47.75 pm/K with increasing temperature. The temperature sensitivity of the sensor is similar to the waveguide Bragg grating sensor presented by Meyer et al., who used the same photoresist for the fabrication of their nonevanescent sensors and achieved a −44 pm/K temperature sensitivity under a 42.5% humidity level [14]. Compared to an integrated eFBG sensor based on silica fiber from Lee et al., which achieved a temperature sensitivity of −13 pm/K, our RWBG sensor shows a 3.7× higher temperature sensitivity performance [10]. On the other hand, compared to the FBG sensor inscribed in a polymer optical fiber presented by Pospori et al., which demonstrated a temperature sensitivity of 21.5 pm/K for an increasing temperature measurement without prestrain, our presented RWBG sensor also shows a 2.2× better performance in temperature sensing [26].

### 3.2. Liquid-Phase Evanescent Field Measurement: Refractive Index Sensitivity

**Figure 5:**
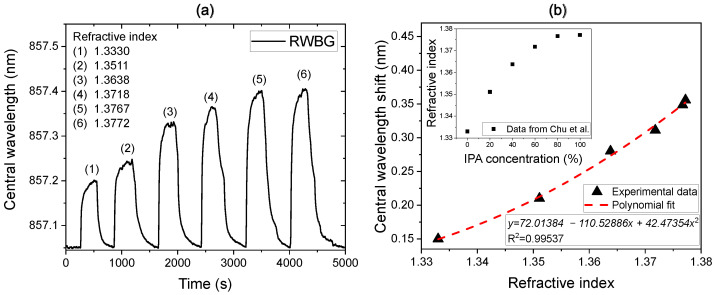
(**a**) Measured RWBG central wavelength shift course when flushed with aqueous solutions with different IPA concentrations. (**b**) Plotted measurement points fitted with a polynomial function, whereby the adopted refractive indices are displayed in the inset.

To validate the functionality of the direct liquid-phase evanescent field measurement, aqueous IPA solutions with different concentrations, ranging from 0 to 99.9%, were successively applied on the RWBG sensor integrated into the LOC device guided by the T-junction microchannel structure. Within the experiment, the aqueous IPA solutions were manually injected into the RWBG sensor chip through one inlet channel with a syringe until the Bragg grating was immersed completely with the test solution. At the same time, the central wavelength shift in the reflection signal was monitored with the optical measurement setup introduced in Figure 3. When the signal stabilization was reached, the liquid was pressed out with air injection so that the monitored Bragg wavelength could return to the original position. The result of this measurement is displayed in Figure 5a.

During the measurement, the monitored shift in the RWBG central wavelength increased noticeably; when the sensor surface was wetted with IPA solutions, the average sensor response time lied at 120 s. The RWBG central wavelength shifts depending on the refractive indexes of the different surrounding aqueous IPA solutions are displayed in Figure 5b; here, we adopted the refractive index of the corresponding IPA solutions from Chu et al. [27], which is additionally shown in the inset of Figure 5b. The overall sensor response during the measurement can be fitted with a polynomial function.

According to the data in Figure 5b, the sensitivity of the sensor is increasing from 2.7 nm/RIU (refractive index unit) for pure water (with 1.3330 RIU) to 6.5 nm/RIU for a pure IPA (with 1.3772 RIU). The increasing sensitivity of the sensor with the increasing refractive index of the surrounding media is caused by the increasing evanescent field volume of the guided light, leaking out of the surface of the waveguide. Considerably, the sensitivity reaches its maximum if the medium has a refractive index close to the refractive index of the waveguide (<1.58). In comparison with the uncoated eFBG sensor from Eisner et al., which possessed a central Bragg wavelength of 852 nm and achieved a sensitivity of 1.33 nm/RIU in 1.33–1.36 RIU, while in 1.4–1.43 RIU, the sensitivity increases to approximately 3.79 nm/RIU [28], our RWBG sensor demonstrates an increased sensitivity.

Furthermore, according to the response time of the RWBG sensor and considering that the overall volume of the T-junction structure is about 7 mm^3^, we are able to estimate the average volume flow rate of the aqueous solutions inside the microchannels to be 0.06 mm^3^/s. Here we assume that the sensor is responding instantaneously, and hence the response time represents the time needed to cover the sensor completely with the injected fluid (until the wavelength shift reaches a stabilized position). In cases of more complicated LOC device structures for specific applications, the local flow rate can depend on different structures in the LOC and may not be measured accurately from the external pump rate. Therefore, this experiment also demonstrates the potential of the RWBG sensor applied for in situ flow-rate monitoring.

We attribute the main effect of the wavelength shift in the aqueous solutions to the evanescent near-field interaction of the guided light close to the surface of the waveguide. On the other hand, some polymers like PMMA absorb water by clustering water within the polymer matrix, which causes the swelling of the polymer. This effect was used by Zhang et al. to measure saline concentrations in water by using a polymer optical fiber with Bragg grating structures [29]. Within their work, the maximum signal (the Bragg wavelength shift) toward longer wavelengths was reached in pure water because then the water content inside the polymer matrix was maximal, leading to the maximal swelling of the fiber. In salt water, their Bragg signal decreased toward shorter wavelengths due to the decreasing water content inside the polymer matrix driven by osmotic pressure. In our measurement, the Bragg wavelength shift is increasing toward longer wavelengths when the concentration of water is decreasing in the IPA solution. In pure IPA (no water), the measured signal shift was the highest. This suggests that water absorption in the polymer matrix of our waveguide plays a minor role and that the evanescent interaction of the guided light at the waveguide’s surface is predominant. However, in the gas phase, humidity can affect our sensor. To investigate the influence of environmental humidity on the polymeric RWBG sensor, we placed an open and uncapped RWBG sensor successively at different humidity levels inside a sealed container for 24 h. At 9% relative humidity (RH), the central wavelength of the RWBG was measured with a decrease of 0.25 nm. While at 75% RH, the central wavelength of the RWBG increased by 0.28 nm. Overall, the sensor showed a wavelength shift under a humidity influence of around 8 pm/% RH. The humidity influence on the sensor is on a longer time scale than the measurements shown in Figure 5. Therefore, since the liquid phase RI measurement was carried out on a much shorter time scale, we assume that the humidity influence on the measurement result is not the dominant effect leading to different central wavelength shifts in response to IPA solutions on the measured RWBG signal.

### 3.3. Gas-Phase Hydrogen Selective Measurement

**Figure 6:**
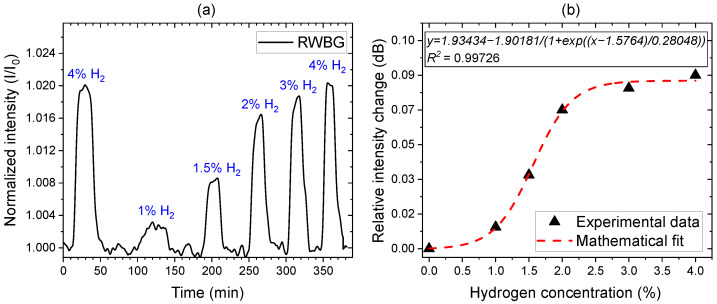
(**a**) Time resolved measurement of different, sequently adjusted hydrogen gas concentrations in the gas chamber. (**b**) Relative changes of light intensity induced by the ambient hydrogen concentration.

For the selective gas-phase hydrogen detection, the RWBG sensor coated with palladium nanoparticles was enclosed in an experimental gas chamber that allows for gas flow regulated with a multigas mass flow controller (see Section 2.5). The total gas flow was kept constant with 100 standard cubic centimeters per minute (sccm) by a multigas mass flow controller for all gas mixtures applied. During the measurement, the RWBG sensor chip was exposed to nitrogen gas mixed with different concentrations of hydrogen up to 4 vol%, and the measurement results are displayed in Figure 6.

At the beginning of the measurement, the gas chamber is flushed with 100 vol% nitrogen for 15 min to ensure a pure environment for the sensing reaction. For each hydrogen measurement cycle, the sensor was exposed to a hydrogen/nitrogen gas mixture, followed by a pure nitrogen gas flush. Figure 6a shows the monitored intensity change in the reflected Bragg grating signal regarding different hydrogen concentrations in nitrogen. During the measurement, the light intensity of the SLED in the measurement setup (see Figure 3) shifted over time; therefore, the measurement results displayed in Figure 6a are corrected by a baseline to compensate for this effect. For each hydrogen level, a different and clear increase in the signal intensity can be observed. The last flushing cycle using a 4 vol% hydrogen concentration again demonstrates the reproducibility of the measurement in comparison to the first cycle. The average response time for the signal intensity to reach a stabilized level lies at 120 s. Throughout the measurement, not only the intensity but also the wavelength shift of the reflected Bragg signal was monitored. However, we could not measure significant changes in the central wavelength of the Bragg signal with increasing hydrogen contents. Without the functional coating of the palladium nanoparticles, we measured no change in the intensity of the reflected Bragg signal.

For a more detailed analysis, the experimental results are plotted in Figure 6b. The plotted measurement results reveal a nonlinear signal intensity shift with increasing hydrogen concentration. Especially at hydrogen concentrations between 1 and 2 vol%, the sensitivity is higher. At 4 vol% hydrogen content, the sensor sensitivity decreases, but the signal intensity shift remains noticeable. For higher hydrogen concentrations above 5 vol%, the sensor response is saturated and is unable to show a higher-intensity shift. The overall intensity changes can be fitted by using a nonlinear mathematical function. A similar nonlinear sensitivity characteristic was also reported in other works, and we assume that it is caused by the α−β phase transition of the palladium nanoparticles [19,30]. Despite the nonlinear response, the RWBG sensor can still be used as a hydrogen indicator.

## 4. Conclusions and Outlook

In our work, we demonstrated a multifunctional RWBG sensor integrated into a LOC device that can measure refractive indices of different concentrated aqueous solutions as well as temperatures at any specific locations inside the LOC. During the measurements, the RWBG sensor achieved a temperature sensitivity of −47.75 pm/K at an increasing temperature from 20 °C to 60 °C as well as a refractive index sensitivity of 6.5 nm/RIU from air to 99.9% pure IPA. On the short time scale, water absorption in the polymer waveguide was not affecting the measurements. When coated with palladium nanoparticles, the sensor achieved a reproducible hydrogen detection up to 4 vol%.

According to the presented measurements, our RWBG sensor shows a promising potential for applications based on the LOC concept. The demonstrated sensing performance for temperature, the refractive index and selective gas measurements can be used in various LOC-based chemical applications like the in situ sensing of temperature or concentration of the liquid solutions in the microchannel. Additionally, with the palladium nanoparticle functional coating, the RWBG sensor chip can be applied for applications like investigating the underground biomethanation concept, indicating the remaining hydrogen level during the methanation process.

However, despite the presented sensitivities of our measurements for various applications, the performance of the sensor can still be influenced by potential cross-sensitivity effects, like changes in temperature. To overcome this, the polymer ridge waveguide can be inscribed with multiple Bragg grating structures in different wavelength areas at different positions, forming a sensor multiplexing array. In this case, different sensors could provide referencing data that compensate for disruptive effects like undesired temperature influences, resulting in an increased sensor accuracy. According to systematic tests of the sensor in different humid atmospheres, the relative humidity (RH) sensitivity was around 8 pm/%RH. The influence of humidity could be neglected in controlled LOC applications or when humidity-insensitive polymer materials are used [31].

## Data Availability

Data will be made available upon request.
